# Fungal Platform for Direct Chiral Phosphonic Building Blocks Production. Closer Look on Conversion Pathway

**DOI:** 10.1007/s12010-014-1356-6

**Published:** 2014-11-16

**Authors:** Ewa Żymańczyk-Duda, Małgorzata Brzezińska-Rodak, Kinga Kozyra, Magdalena Klimek-Ochab

**Affiliations:** Department of Bioorganic Chemistry, Faculty of Chemistry, Wroclaw University of Technology, Wybrzeże Wyspiańskiego 27, 50-370 Wrocław, Poland

**Keywords:** Chiral aminophosphonates, *Rhodospirillum*, Biocatalysis, Deracemisation, Yeast transformation

## Abstract

The application of *Rhodospirillum toruloides* strain allowed resolving the chemically synthesized racemic mixtures of following chiral aminophosphonic acids: 1-aminoethylphosphonic acid (1), 1-amino-1-*iso*-propyl-1-phosphonic acid (2), 1-amino-1-phenylmethylphosphonic acid (4) and 1-amino-2-phenylethylphosphonic acid (3). The applied protocols resulted in obtaining pure (*R*)-1-aminoethylphosphonic acid (100 % of e.e.) and enantiomerically enriched mixtures of other phosphonates (73 % e.e. of (*S*)-1-amino-1-phenylmethylphosphonic acid, 51 % e.e. of (*R*)-1-amino-2-phenylethylphosphonic acid and 40 % e.e. of (*S*)-1-amino-2-methylpropylphosphonic acid). Products are valuable chiral building blocks and serve as aminophosphonic acids platform for further applications. Performed experiments allowed to define the path of xenobiotics bioconversion.

## Introduction

**Fig. 1:**
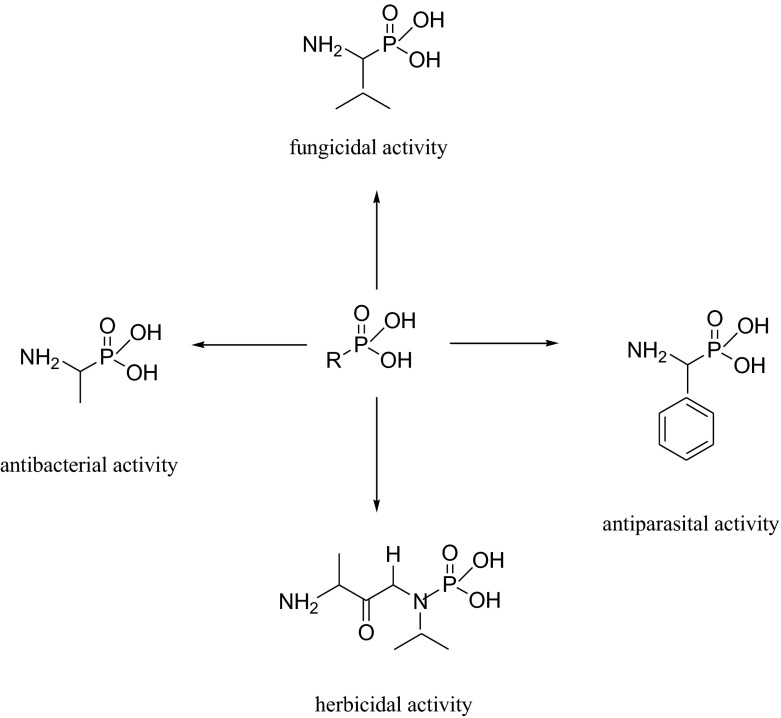
Biologically active phosphonates [16–18]

Aminophosphonic acids are organophosphorus compounds of variable biological activities as indicated before [1, 2]. Although phosphonic and carboxylic acid differ considerably with respect to shape, size and acidity, α-aminophosphonic acids are considered as structural analogues of amino acids or as mimetics of the transition state of enzymatic peptide bond hydrolysis. As a consequence, they exhibit inhibitory activity against different enzymes, especially towards proteinases such as the HIV protease, thrombin, aminopeptidases and human collagenase [3–5]. Additionally, aminophosphonic acids are also applied in hydrometallurgy for metal extraction and in diagnostic medicine as screening agents [6]. As it is known, particular biological activity of specific chiral compound is usually related to its absolute configuration [7]. On account of different biological and chemical applications of phosphonates (Fig. 1), development of effective synthetic methodologies for their preparation in optically pure forms has been a matter of interest for several research groups and is still not a fully explored field of science [8–15].

Biocatalysis has been successfully used for synthesis of pure enantiomers of organophosphonates derivatives and it represents an alternative for expensive asymmetric organic synthesis [19–27]. *Rhodospirillum toruloides* is known as a valuable source of enzymes. This strain is able to utilize natural amino acids and their synthetic analogues or derivatives via, among others, oxidative deamination, thanks to the activity of amino acids oxidases (LAAO or DAAO) [28–31]. Presented article is a continuation of our previous work and reports successful application of the whole-cell biocatalyst—*R. toruloides* strain for the enantiomeric enrichment of chemically synthesized racemic mixtures of chiral α-aminophosphonic acids. The presupposition of such approach was that the racemic mixtures of chosen substrates (aminophosphonates) are resolved via catabolic pathways, which are involved in amino acids metabolism in *R. toruloides* cells. Taking this into account, in some experiments, natural amino acids in optically pure forms were used as chemical additives to initiate the bioprocess, what was followed by oxidases activity assignment. The results of such approach were as a matter of fact very surprising and in contradiction with presuppositions, what is discussed through the text body.

## Materials and Methods

### Substrates Synthesis and Characteristic

α-Aminophosphonic acids were synthesized according to the method described in literature [32]. Chemical structures were confirmed by ^1^H and ^31^P NMR spectroscopy. Aminophosphonic acids were purified by column, ion exchange chromatography (Dowex 50X8, 200–400 mesh; eluted with water) or by recrystallisation (ethyl alcohol/water (*v/v* 3:1)).

Spectroscopic data were as follows:

- 1-aminoethylphosphonic acid (1): Yield, 38 %; 31P NMR δ (ppm), 14.78; 1H NMR δ (ppm), 1.28 (dd, J = 6 Hz, 3H, CH_3_), 3.20 (m, 1H, CHP);
- 1-amino-1-*iso*-propyl-1-phosphonic acid (2): Yield, 27 %; 31P NMR δ (ppm), 13.44; 1H NMR δ (ppm), 0.95 (dd, J = 6.9 Hz, 6H, (CH_3_)2CH), 2.00–2.15 (m, 1H, (CH_3_)2CH), 2.94 (dd, J = 6.3 Hz, 1H, CHP);
- 1-amino-2-phenylethylphosphonic acid (3): Yield, 21 %; 31P NMR δ (ppm), 11.51; 1H NMR δ (ppm), 2.58–2.77 (m, 1H, CHP), 3.34–3.80 (m, 2H, CH_2_), 7.14–7.36 (m, 5H, Ph);
- 1-aminophenylmethylphosphonic acid (4): Yield, 24 %; 31P NMR δ (ppm), 10.60; 1H NMR δ (ppm) 4.19 (d, J = 15.5 Hz, 1H, CHP), 7.29 (s, 5H, Ph).

### Microorganism Cultivation

Cultivation without amino acid supplementation: *Rhodosporidium toroluides* strain DSM 4444 was cultivated on the recommended DSMZ Medium 90: malt extract (30 g), soya peptone (3 g) dissolved in distilled water (1000 mL), pH 5.6 (100 mL in 250-mL cultivation flasks, with shaking 100 rpm at room temperature).

Cultivation with the amino acid supplementation: microorganisms were cultivated on the modified malt medium consisted of: malt extract (15 g), yeast extract (10 g) and 0.1 g of chosen amino acid (*d*-alanine or *l*-leucine or *d*-methionine) dissolved in distilled water (1000 mL)) [33–35].

After cultivation, biomass was separated by centrifugation (8000 rpm/10 min), and 1.5 g of wet cells (from every single cultivation flask) was used as a biocatalyst (after 4 days of growing) or cells were disintegrated to enzymatic activity assignment (after 2–5 days of cultivation).

### Disintegration Procedure

Fresh wet cells (1.5 g) were washed with water and then suspended in 15 mL of a cold phosphate buffer (100 mM, pH 8.3, T = 4 °C) with β-mercaptoethanol (5 mM), 2 mM EDTA and 0.1 % Triton X-100 and mechanically disintegrated with sea sand for 15 min using a cooling bath (ice/water). After that, crude cells extract was centrifuged (11,000 rpm, 4 °C, 10 min), and the supernatant was analysed.

The efficiency of cell disruptions was monitored by measurement of the concentration of the soluble proteins in the cell-free extract. Each experiment was done in triplicate.

### Protein Concentration Assay

Soluble protein concentrations in clarified supernatants were estimated by the Bradford method using bovine serum albumin as a standard [36].

### Enzyme Activity Assay

All assays were done in air-saturated solutions at 30 °C according to the peroxidase/o-dianisidine method. The activity of the intracellular enzymes (L/D amino acid oxidases) was determined by the method described by Fisher G. [35]. Absorbance was measured at 436 nm over a period of 1–10 min on UV/Vis spectrometer (LAMBDA Bio, PerkinElmer). Total activity of the oxidases in the crude extracts was given as a specific activity of the enzyme and was expressed as units per milligram of the total amount of proteins (U mg^−1^).

### Biotransformation—Initial Procedure

Ten milligram of substrate: 1-aminoethylphosphonic acid (1), 1-amino-1-*iso*-propyl-1-phosphonic acid (2), 1-amino-1-phenylmethylphosphonic acid (3) or 1-amino-2-phenylethylphosphonic acid (4) (Fig. 1.) and 1.5 g of wet cells (after cultivation with/without amino acids supplementation) were added to 250-mL Erlenmeyer flasks, containing 50 mL of phosphate buffer (0.017 mol L^−1^, pH 6.11). Biocatalysis was carried out for 1–7 days with shaking at 250 rpm. Subsequently, cells were removed by centrifugation (8000 rpm, 10 min), supernatant was evaporated under reduced pressure and crude products were analysed by means of ^31^P NMR spectroscopy to direct confirmation of bioconversion. This allowed choosing effective duration of the process—4 days of bioconversion (considering also the enzymatic activity assignment). Experiments were done in triplicate.

### Preparative Biotransformation

The 0.24 mmol of the particular substrate and 4.5 g of wet cells (after cultivation with/without amino acids supplementation) were added to 500-mL Erlenmeyer flasks, containing 200 mL of phosphate buffer (0.017 mol L^−1^, pH 6.11) and shacked at 250 rpm for 4 days. Then, cells were centrifuged (8000 rpm, 10 min), collected supernatants were evaporated under reduced pressure and crude products were purified by ion exchange chromatography (Dowex 50X8, 200–400 mesh; eluted with water) and analysed by means of ^31^P NMR spectroscopy. Simultaneously, control cultivations (with cells without substrate and with substrate without the biocatalyst) were also performed.

### Derivatisation of Enantiomerically Enriched Mixture of 1-Aminoethylphosphonic Acid

Tosyl derivative of 1-aminoethylphosphonic acid was prepared by heating the crude biotransformation product (0.12 mmol) in 10 mL of 0.4 mol L^−1^ phosphate buffer (pH 11) with 70 mg of TsCl in 10 mL of acetonitrile at 50 °C for 30 min [37]. Then, the mixture was extracted twice with ethyl acetate, followed by evaporation under reduced pressure of volatile components, and then ^31^P NMR analysis was performed. Tosylated 1-aminoethylphosphonic acid: ^31^P NMR δ (ppm): 26.1; mass spectroscopy (Spectrometer: LCT Premier XE Waters with ESI+ ionisation): sodium adduct 302.0231 (theoretical: 302.0228), dimer—sodium adduct 581.0445.

### ^31^P NMR Assignments

NMR spectra were recorded on Bruker Advance TM instrument operating at 600 MHz or spectrometer Bruker Advance DRX 300 operating at 300 MHz; measurements were made in deuterium oxide at temperature of 300 K.

### Enantiomeric Excess Assignments

Enantiomeric composition of products was evaluated using ^31^P NMR technique. Spectra were recorded with the addition of α-cyclodextrin (chiral solvating agent [38] and biotransformation product (respectively 2:1 *w/w*). Optical purity of 1-aminoethylphosphonic acid (1) was assigned for its tosylated derivative. The pH of the samples was set at 10–11; excluding samples prepared for 1-amino-1-phenylmethylphosphonic acid analysis, in which pH value was set at 2.

### Assignment of Absolute Configuration of Predominant Enantiomer

Absolute configurations of biotransformation products were determined by measurements of the optical rotation (in 1 M NaOH, c = 1) [9, 19, 39, 40]. Specific rotations (calculated from % of e.e. computed using ^31^P NMR spectra and measured optical rotation values) of pure enantiomers were as follows: for (*R*) 1-aminoethylphosphonic acid (1), [*α*]_578_ = −15.4°; for (*S*)-1-amino-1-*iso*-propyl-1-phosphonic acid (2), [*α*]_578_ = − 0.97°; for (*R)*-1-amino-2-phenylethylphosphonic acid (3), [*α*]_578_ = − 47.9°; and finally, for (*S*)-1-amino-1-phenylmethylphosphonic acid (4), [*α*]_578_ = −17.5°.

## Results and Discussion

**Fig. 2:**
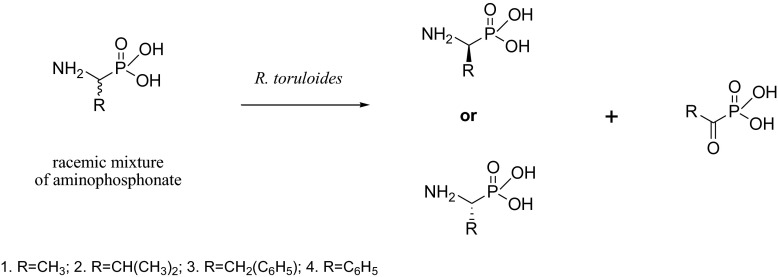
Kinetic resolution of racemic mixtures of aminophosphonic acids by *R. toruloides*—possible mechanisms

Chiral aminophosphonic acids of defined absolute configuration are both of natural origin—they exist in living systems and of artificial origin—synthetic ones—received for a specific purpose, which is usually connected with their broad possible spectrum of biological activity [2]. Steric structure of these compounds is crucial for their further applications; so, pursuit for new active compounds of this kind or new synthetic methods of obtaining them is still of considerable importance [41–47]. An expensive, asymmetric synthesis of such structures requires aldehydes, ketones or carboxylic acids as substrates and frequently resulted in quite moderate productivities and enantiopurities [6, 48, 49]. Thus, efficient biotransformation appears to be a good alternative. The aim of this work was to elaborate the protocol—as simple as possible—allowing bioresolution of the racemic mixtures of chemically synthesized aminophosphonates of different structures with the use of *R. toruloides* cells. Bearing in mind the structural analogy of these substrates to amino acids, it was reasonable to expect the similar path of bioconversion of applied xenobiotics, which in this case usually starts from oxidative deamination of the substrate (Fig. 2). The experimental efforts resulted in enantiomeric enrichment of the mixtures of chiral substrates (Table 1) and allowed to speculate what is the first reaction; the effect of which is observed as deracemisation.

**Table 1:** Results of the resolution of racemic mixtures of aminophosphonic acids by *R.toruloides*

Substrate (according to Fig. 2)	Conversion (%)	Enantiomeric excess (%)	Absolute configuration
1	50	100	R
2	20	40	S
3	25.5	51	R
4	36.5	73	S

Biotransformation was carried out according to preparative procedure; biocatalyst was cultivated without supplementation

**Fig. 3:**
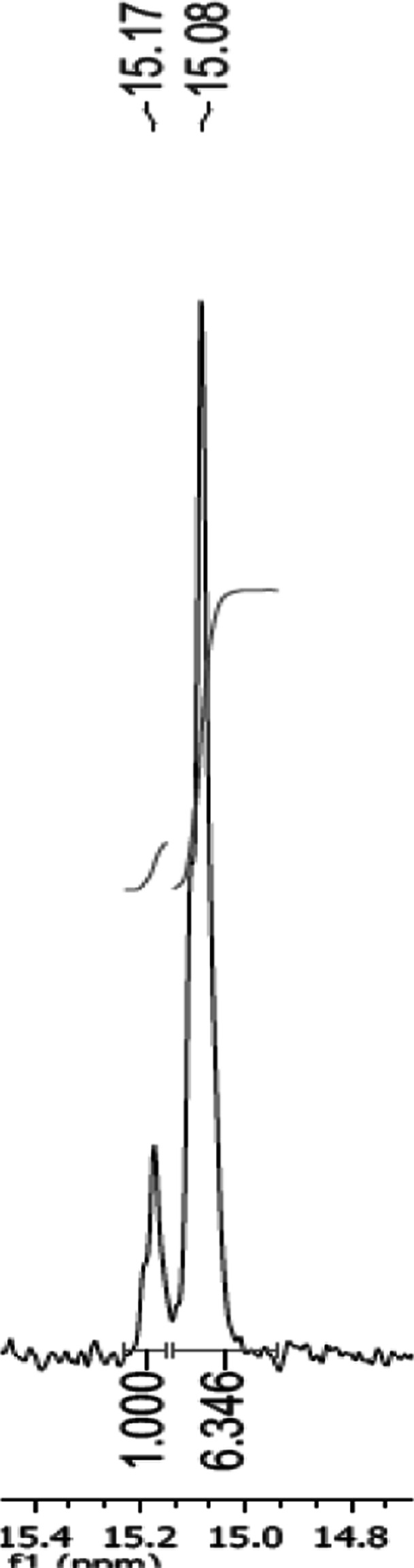
^31^P NMR spectrum recorded with α-cyclodextrin after biotransformation of 1-amino-1-phenylmethylphosphonic acid—predominant enantiomer is defined as *S*

^31^P NMR spectra, recorded after the completion of the process, indicated that deracemisation can be achieved in two ways: either by deamination with ketophosphonic acid synthesis (Fig. 2) or with complete mineralisation of one from the pair of starting enantiomers. It was important to define some conditions, which determined the route of bioconversion paths, because there was a possibility of further bioconversion of obtained ketophosphonates into corresponding chiral hydroxy derivative. Thus, such two-step process was recorded for 1-aminoethylphosphonate (1) with, unfortunately, very low final productivity, what made the isolation of the product—hydroxyphosphonate—impossible (data not shown). The most effective deracemisation was obtained also for compound 1 (100 % of (*R*)). This substrate—alanine structural analogue—probably passes the cell envelopes easier than others, without any structural hindrance, what explains its oxidative deamination followed by inside cell reduction into hydroxy derivative. It is known from the literature data that fungi seemed to be quite individualistic in the organisation of their amino acid transport abilities [50]. The absolute configuration of the nonreactive enantiomer is an additional element, which confirms the oxidative path of bioconversion. Thus, the *R. toruloides* strain metabolizes *d*-Alanine (*S*-Ala) into the corresponding α-keto acid and ammonia [51], what is in agreement with the experimental data, where (*S*) aminophosphonic acid is bioconverted, whereas (*R*)—enantiomer is isolated as valuable product. The result of bioconversion of 1-aminoethylphosphonate delivers also another indirect evidence for such mechanism of biotransformation—the lack of the reaction was observed in the case of *R. toruloides* cells cultivated with the supplementation step (data not shown). It suggests that *d*-alanine added as the supplement is probably partially accumulated in the cells and, as a consequence, it competes with xenobiotic substrate either for transport system or active site of enzymes involved in discussed process. This puzzling aspect of amino acid transport has been reported for fungal cells and called transinhibition. This was the inhibition of transport of one amino acid by preincubation of the fungus with another amino acid. Since the fungus was washed free of the first amino acid, the inhibition was not the results of competition for transport at the cell surface but appeared to be a result of interference with transport by high internal concentration [50]. This phenomenon would allow a regulation of the intracellular amino acid pools and prevent deleterious cytosolic amino acid concentrations in the fungal cell [52]. Satisfactory results were achieved also for the resolution of racemic mixture of substrate 4 (1-amino-1-phenylmethylphosphonic acid)—enantiomeric enrichment reached 73 % of e.e—see representative NMR spectrum (Fig. 3).

This time also, the effectiveness of the process was satisfactory only after the application of biocatalyst cultivated without the amino acid supplementation. This allowed concluding that in general: the supplementation during *R. toruloides* growing step decreases the productivity of biocatalysed kinetic resolution of racemic mixtures of chiral aminophosphonates. This was the most surprising uncover of presented work and the results of deracemisation carried out according to procedure without induction step, ranged from the lack of the reaction for substrate 1 to the maximum 33 % of e.e for *R* enantiomer of substrate 3 (1-amino-2-phenylethylphosphonic acid). Experimental data (not shown) proved that the highest *d*-amino acid oxidase activity (11.46 U mg^−1^) was observed after 4 days of *R. toruloides* incubation with the addition of *d*-Ala as a source of organic nitrogen or/and carbon. Moreover, without amino acids supplementation or with *l*-amino acids addition, measured enzymatic activity dramatically decreases 2.2 U mg^−1^ and with *l*-leucine—2.9 U mg^−1^, respectively. These results confirmed that *d*-amino acid oxidase belongs to a group of inducible enzymes, whereas *l*-amino acid oxidase induction failed. This allowed to assume that aminophosphonic acids act themselves as oxidases inductors. What is also important is that they are able to stimulate the yeast cells to synthetize the oxidases of opposite enantiospecificity, what explains the differences in absolute configurations of predominant enantiomers obtaining as products (Table 1). This seems to be in agreement with the limited literature data about the *l*-amino acid oxidases, which play an important role in the innate immune defences of animals and have antimicrobial features—antibacterial activity in the mammary glands and are synthesized inside the living cells only under stress conditions, e.g. in the presence of non-physiological compounds in the environment, without the necessity of induction [53]. Differences in the efficiency and stereospecificity of the same biocatalyst towards the particular substrates can result from the phosphonate features as far as they are able to interact with the native enzymes involved in their conversion locking out the specific enzymatic activities. Work with phosphonates always requires the maintenance of the delicate balance between their inhibitory features (the values of concentrations are limited) and the efforts to increase the productivity of the method.

In conclusion, a new and simple method of enantiomeric enrichment of chemically synthesized racemic mixtures of chiral aminophosphonates was elaborated. Viable cells of *R. toruoides* strain appeared to be an effective tool for gaining of enantiomers of chiral xenobiotics for further possible applications.
